# Wie altersfreundlich sind Städte und Gemeinden? Deutsche Version des Age-Friendly Cities and Communities Questionnaire (AFCCQ)

**DOI:** 10.1007/s00391-025-02440-6

**Published:** 2025-04-30

**Authors:** Adele Grenz, Michael Weinhardt, Moritz Hess, Joost van Hoof, Jeroen Dikken, Kathrin Boerner

**Affiliations:** 1 Abteilung Präventions- und Rehabilitationsforschung, Department für Versorgungsforschung, Fakultät VI Medizin und Gesundheitswissenschaften, Carl von Ossietzky Universität Oldenburg, Oldenburg, Deutschland; 2 Deutsches Zentrum für Altersfragen, Berlin, Deutschland; 3 Fachbereich Sozialwesen, Hochschule Niederrhein, Krefeld, Deutschland; 4 Research Group of Urban Ageing, Faculty of Social Work & Education, The Hague University of Applied Sciences, Den Haag, Niederlande; 5 Department of Systems Research, Faculty of Spatial Management and Landscape Architecture, Wrocław University of Environmental and Life Sciences, Wrocław, Polen

**Keywords:** Urbanisierung, Demografischer Wandel, Altersfreundlichkeit, Stadtplanung, Fragebogen, Urbanisation, Demographic Change, Age-Friendliness, Urban Planning, Questionnaire

## Abstract

**Hintergrund und Ziel:**

Urbanisierung und demografischer Wandel stellen soziale Herausforderungen dar, die in der Stadt- und Versorgungsplanung oft unzureichend berücksichtigt werden. Zur Messung von Altersfreundlichkeit wird in dieser Studie eine deutschsprachige Version des „Age-Friendly Cities and Communities Questionnaire“ (AFCCQ) vorgestellt. Ziel ist es, die Altersfreundlichkeit aus Sicht älterer Menschen zu erfassen und unterschiedliche Bedarfe in Städten zu identifizieren. Zudem werden Ergebnisse für die Stadt Oldenburg präsentiert.

**Methode:**

Die Validierungsstudie wurde mit einer repräsentativen Stichprobe älterer Personen in Oldenburg (*n* = 905) durchgeführt. Die Teilnehmenden beantworteten den Fragebogen AFCCQ-DE mit 23 Items und bewerteten ihre Erfahrungen in der Stadt auf einer 5-stufigen Likert-Skala (Stimme überhaupt nicht zu - Stimme voll und ganz zu). Anschließend wurden eine Faktorenanalyse und eine Clusteranalyse durchgeführt.

**Ergebnisse:**

Die psychometrische Validität und Reliabilität des AFCCQ-DE konnte bestätigt werden. Der erstmalig ins Deutsche übersetzte Fragebogen erwies sich als robustes Instrument, das nun im deutschsprachigen Raum eingesetzt werden kann. Unter Einsatz clusteranalytischer Verfahren wurden vier Gruppen älterer Menschen identifiziert, die sich hinsichtlich ihrer Personenmerkmale und Bedarfe systematisch unterscheiden.

**Diskussion:**

Die Ergebnisse betonen die Bedeutung eines standardisierten Instruments wie des AFCCQ für die Planung und Bewertung der Altersfreundlichkeit von Städten und Gemeinden. Die gewonnenen Daten bieten eine Grundlage für die Entwicklung zielgerichteter Stadtplanungsmaßnahmen, die die Bedürfnisse älterer Menschen gezielt ansprechen und gutes Altern in urbanen Räumen unterstützen.

**Zusatzmaterial online:**

Zusätzliche Informationen sind in der Online-Version dieses Artikels (10.1007/s00391-025-02440-6) enthalten.

## Abstract (en)

**Background and objective:**

Urbanisation and demographic change present social challenges that are often insufficiently addressed in urban and care planning. This study introduces a German-language version of the Age-Friendly Cities and Communities Questionnaire (AFCCQ) as a tool for measuring age-friendliness. The aim is to assess age-friendliness from the perspective of older adults and to identify varying needs within cities. In addition, findings for the city of Oldenburg are presented.

**Method:**

The validation study was conducted with a representative sample of older people in Oldenburg (*n* = 905). The participants completed the AFCCQ-DE questionnaire, consisting of 23 items, and rated their experiences in the city using a 5-point Likert scale (strongly disagree – strongly agree). A factor analysis and a cluster analysis were then carried out.

**Results:**

The psychometric validity and reliability of the AFCCQ-DE were confirmed. The questionnaire, which was translated into German for the first time, proved to be a robust instrument that can now be used in German-speaking countries. Using cluster-analytical methods, four groups of older people were identified, differing systematically in terms of their personal characteristics and needs.

**Discussion:**

The results highlight the importance of a standardised instrument such as the AFCCQ for planning and evaluating the age-friendliness of cities and municipalities. The collected data provide a basis for the development of targeted urban planning measures that specifically address the needs of older adults and support ageing well in urban environments.

## Hintergrund

Urbanisierung und demografischer Wandel verändern weltweit die Lebensbedingungen. Bis 2050 könnten 84,3 % der Menschen in Städten leben. In Deutschland wohnen derzeit etwa 40,5 % Menschen in Städten und Ballungsräumen [1]; parallel dazu wird die Bevölkerung im Mittel kontinuierlich älter. Bis 2050 wird sich die Anzahl der über 80-Jährigen voraussichtlich fast verdreifachen [2]. Diese demografischen Veränderungen prägen zunehmend das Stadtbild und stellen Stadtentwicklung und kommunale Planungen vor neue Herausforderungen. Menschen möchten in ihrem gewohnten Umfeld alt und versorgt werden und haben dabei unterschiedliche Bedarfe [3]. Dies steht im Spannungsfeld zu Verfügbarkeit sowie Zugänglichkeit von Unterstützungsangeboten, Dienstleistungen und informellen Hilfen im sozialen und im gesundheitlichen Bereich. Studien zeigen, dass altersfreundliche Umfelder mit körperlicher [4, 5] mentaler [6, 7] und sozialer [8–13] Gesundheit assoziiert sind.

Die Weltgesundheitsorganisation (WHO) startete 2010 das Netzwerk „Age-friendly Cities and Communities“ mit dem Ziel, älteren Menschen ein aktives, selbstbestimmtes Altern zu ermöglichen und ihr Wohlbefinden durch soziale und wirtschaftliche Teilhabe zu fördern. Als relevant für die Altersfreundlichkeit gelten 8 Bereiche: 1) Wohnen; 2) soziale Teilhabe; 3) Respekt und soziale Eingliederung; 4) Bürgerbeteiligung und Beschäftigung; 5) Kommunikation und Information; 6) gemeinschaftliche Unterstützung und Gesundheitsdienste; 7) öffentlicher Raum und Gebäude; 8) Verkehr.

Allerdings besteht die Herausforderung, Altersfreundlichkeit valide zu erheben und zu quantifizieren. In den letzten Jahren wurden verschiedene methodische Ansätze entwickelt, um Altersfreundlichkeit aus Sicht der Bürger*innen zu messen [14–16]. Bei existierenden Erhebungsinstrumenten fehlten häufig relevante Informationen zum Entwicklungs- und zum Validierungsprozess, insbesondere zur Konstruktvalidierung und der genauen Einbindung der 8 WHO-Bereiche. Zudem gab es einen Mangel an handhabbaren, langfristig einsetzbaren Instrumenten [16]. Daher bestand Bedarf an quantitativen, interkulturell vergleichbaren Messmethoden [17].

Der Fragebogen „Age-Friendly Cities and Communities Questionnaire“ (AFCCQ) wurde ursprünglich in den Niederlanden [16] entwickelt und ist ein standardisiertes, validiertes Instrument zur Erfassung der Altersfreundlichkeit von Städten und Gemeinden. Das Instrument umfasst 23 Items, die alle WHO-Bereiche und zusätzlich die wahrgenommene finanzielle Situation abdecken, und folgt in der Entwicklung dem COSMIN-Protokoll [18]. Die Bewertungen zu 9 verschiedenen Lebensbereichen älterer Menschen ab 65 Jahren werden über eine 5‑stufige Likert-Skala (Stimme überhaupt nicht zu bis Stimme voll und ganz zu) erfasst. In deutscher Sprache gab es bis dato noch keinen vergleichbaren Fragebogen.

Die vorliegende Studie füllt diese Lücke und stellt eine Übersetzung, transkulturelle Adaptation, psychometrische Evaluation und Cluster-Analyse einer deutschen Version des AFCCQ vor, mit dem Ziel, die Altersfreundlichkeit von Städten und Gemeinden aus der Perspektive älterer Personen zu erfassen und unterschiedliche Bedarfe in städtischen Kontexten zu identifizieren. Zusätzlich werden die Ergebnisse zur Altersfreundlichkeit der Stadt Oldenburg vorgestellt.

Die Entwicklung des AFCCQ in Deutschland ist ein wichtiger Schritt für das kommunale Monitoring [19] von Altersfreundlichkeit, aber auch für die nationale [20] und internationale [21, 22] Vergleichbarkeit der Altersfreundlichkeit von Städten und Gemeinden.

## Methode

**Abb. 1:**
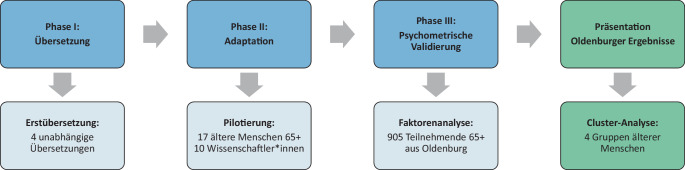
Phasen der Validierung des AFCCQ-DE

Die Validierung der deutschen Übersetzung des AFCCQ wurde in Oldenburg, einer Großstadt im Nordwesten Deutschlands mit rund 172.900 Einwohner*innen, als Querschnittstudie durchgeführt. Oldenburgs demografische Struktur umfasst 13,3 % der Bevölkerung im Alter von 65 bis 79 Jahren (Deutschland: 15,0 %) und 6,3 % ab 80 Jahren (Deutschland: 7,3 %). Die wachsende Stadt, geprägt von einer heterogenen sozioökonomischen Dynamik, weist einen leicht unterdurchschnittlichen Anteil an über 80-Jährigen auf. Dennoch stellen die hohe Einwohnerdichte und der große Anteil an Einpersonenhaushalten besondere Herausforderungen für die Infrastruktur dar, etwa bei der Sicherung von bedarfsgerechtem Wohnraum und der Lebensqualität älterer Menschen [23]. Den Studienverlauf zeigt Abb. 1. Sämtliche statistischen Analysen wurden mit SPSS Version 29.0 durchgeführt.

### Phase 1: Übersetzung

Die Fragebogenübersetzung erfolgte durch die Autor*innengruppe (A.G., K.B., M.W., M.H.) mit ausgewiesener Expertise in der englischen und deutschen Sprache sowie auf den Gebieten der Gerontologie, Psychologie und Soziologie [18]. Zunächst erfolgten 4 unabhängige Übersetzungen der britisch-englischen Version des AFCCQ-EN in die deutsche Sprache. Anschließend wurden die Erstversionen bezüglich kultureller und sprachlicher Diskrepanzen diskutiert und angepasst. Hierbei wurden auch der niederländische Originalfragebogen und die Muttersprachler*innen im Team interpretativ unterstützend hinzugezogen.

### Phase 2: Adaptation

Der Fragebogen wurde in einer Pilotphase anhand eines Convenience Sample durch 17 ältere Personen (65+ Jahre) und 10 Expert*innen aus den Bereichen Gerontologie, Soziale Arbeit und Stadtplanung auf Augenschein- und Inhaltsvalidität geprüft. Die Relevanz der 23 Items wurde auf einer Likert-Skala bewertet und der Item-Inhaltsvalidität-Index (I-CVI) berechnet. Ein I‑CVI-Wert von mindestens 0,78 wird in der Literatur als exzellenter Grenzwert für Inhaltsvalidität angesehen [24, 25]. Freitextkommentare ergänzten die Bewertung, und die erste Version wurde entsprechend überarbeitet.

### Phase 3: Psychometrische Validierung

#### Datenerhebung und Teilnehmende

Das Bürgerbüro zog aus dem Melderegister der Stadt Oldenburg eine Zufallsstichprobe von 2000 in Oldenburg lebenden Personen im Alter von 65 Jahren und älter, die in Privathaushalten wohnen. Diese erhielten im Juli 2024 postalisch den Fragebogen mit frankiertem Rückumschlag und optionalem Online-Zugang. Die Ethik-Kommission der Universität Oldenburg erteilte die Zustimmung zur Fragebogenerhebung (Aktenzeichen: 2024-073, Beschluss vom 21.05.2024).

Von den 905 Studienteilnehmenden (Antwortrate 45 %) füllten 90 % die Papierversion des Fragebogens aus, 10 % antworteten online. Frauen waren mit 54,6 % etwas stärker vertreten als Männer (45,4 %). Die größte Altersgruppe waren die 65- bis 74-Jährigen (49,8 %); ein Drittel war 80 Jahre oder älter. Etwa 50,8 % der Befragten wiesen einen mittleren, 43 % einen hohen und 6,2 % einen niedrigen Bildungsgrad auf (nach ISCED – International Standard Classification of Education). Die meisten (93,2 %) waren in Deutschland geboren, 68,5 % lebten im Eigenheim, 65,3 % mit mindestens einer weiteren Person. Unterstützung im Alltag erhielt ein Drittel (31,9 %), 17,9 % nutzten Rollstuhl oder Rollator. Knapp die Hälfte (49,5 %) gab mindestens eine chronische Erkrankung an. Die Befragten bewerteten ihre selbst eingeschätzte Lebensqualität auf einer Skala von 1 bis 10 im Durchschnitt mit 7,6.

#### Faktorenanalyse

Das Ziel dieser Phase war, ähnlich wie in anderen Ländern, die Überprüfung der Konstruktvalidität des AFCCQ-DE. Die Faktorenstruktur des Fragebogens AFCCQ-DE wurde mit einer konfirmatorischen Faktorenanalyse (CFA) überprüft, um festzustellen, wie gut die deutschen Daten zum ursprünglichen niederländischen Modell [16] passen. Die interne Konsistenz wurde mithilfe der Kompositreliabilität untersucht (Zusatzmaterial online: Sup3_Tab3).

#### Cluster-Analyse

Um Gruppen älterer Menschen mit ähnlichen Merkmalen zusammenzufassen, wurde eine Cluster-Analyse (Zusatzmaterial online: Sup3_Tab4,5) in 2 Stufen durchgeführt [26]. Die erste Stufe diente der Ermittlung der optimalen Cluster-Anzahl (HCA), unter Einsatz der Ward-Methode und des quadrierten euklidischen Abstands; die zweite Stufe verfeinerte die Cluster-Zuordnung („k-means“). Die Cluster-Bildung erfolgte anhand der 9 Bereiche des AFCCQ. Anschließend wurden soziodemografische Merkmale innerhalb und zwischen den Clustern analysiert.

## Ergebnisse

### Phase 1: Übersetzung

Die Erstübersetzung des AFCCQ wurde systematisch durchgeführt. Diskussionen und Anpassungen zielten darauf ab, die Items sprachlich und kulturell verständlich und zugleich präzise zu gestalten. Dabei wurde u. a. der Begriff „neighbourhood“ ausführlich diskutiert. Zur Auswahl standen „Nachbarschaft“, „Wohnviertel“ und „Stadtteil“. Wir entschieden uns für „Wohnviertel“, da dieser Begriff die räumliche Dimension des Alltags treffend beschreibt. Transkulturelle Anpassungen wurden nur wenige vorgenommen (Zusatzmaterial online: Sup2).

### Phase 2: Adaptation

28 Personen bewerteten die Item-Relevanz: Von 23 Items erzielten 21 für die Augenscheinvalidität (17 Personen ab 65 Jahren) und 14 für Inhaltsvalidität (10 Expert*innen) exzellente I‑CVI-Werte (> 0,78). 16 Items wurden noch einmal sprachlich überarbeitet.

### Phase 3: Psychometrische Validierung

In der Oldenburger Studie (*n* = 905) konnte die faktorielle Struktur für die 9 Bereiche des AFCCQ bestätigt werden (Zusatzmaterial online: Sup3_Tab2). Der AFCCQ-DE zeigte in allen Bereichen eine gute Reliabilität (Zusatzmaterial online: Sup3_Tab3) und kann daher als gültig und zuverlässig für den Einsatz im deutschsprachigen Raum angesehen werden (Zusatzmaterial online: Sup1).

#### Altersfreundlichkeit in Oldenburg

Die Gesamtbewertung der Altersfreundlichkeit in Oldenburg lag im leicht positiven Bereich (Tab. 1). Der normalisierte Gesamt-Score wurde gebildet, indem die Rohwerte der 9 Bereiche summiert und auf eine Skala von −10 bis 10 bzw. −90 bis +90 umgerechnet wurden. Die höchsten Bewertungen entfielen auf die Bereiche *Wohnen, Respekt und soziale Einbindung* und *öffentliche Verkehrsmittel*, während *kommunale Unterstützung und Gesundheitsversorgung* sowie *öffentlicher Raum und Gebäude* am niedrigsten bewertet wurden.

**Tab. 1:** Normalisierte Gesamtbewertungen pro Bereich (−10 bis +10)

Bereich	Mittelwert (SD)	Bewertung*
Wohnen	7,1 ± 3,3	+++
Soziale Teilhabe	3,6 ± 3,9	++
Respekt und soziale Einbindung	5,0 ± 4,5	+++
Bürgerschaftliches Engagement und Beteiligung	3,9 ± 3,9	++
Kommunikation und Information	3,8 ± 4,0	++
Kommunale Unterstützung und Gesundheitsversorgung	2,7 ± 3,8	++
Öffentlicher Raum und Gebäude	2,9 ± 4,3	++
Öffentliche Verkehrsmittel	5,1 ± 4,3	+++
Finanzielle Situation	3,6 ± 5,3	++
**AFCCQ-Total (−90 bis +90)**	**38,2 ± 23,6 **	++

*Das Bewertungsschema orientiert sich an der Interpretationshilfe von Dikken et al. (2020) und stellt die Ausprägung der Zufriedenheit (*Plus-Zeichen*) oder Unzufriedenheit (*Minus-Zeichen*) der Altersfreundlichkeit dar [16]. Die Skala reicht von „extrem unzufrieden“ (−−−−) bis „extrem zufrieden“ (++++), die Mitte (−/+) entspricht einer neutralen Bewertung (Zusatzmaterial online: Sup1)

#### Cluster-Analyse

Altersfreundlichkeit wird in der Stadt auf vielfältige Weise erlebt. Die Cluster-Analyse ergab 4 Gruppen älterer Menschen, die sich anhand ihrer Bewertung der Altersfreundlichkeit (Zusatzmaterial online: Sup3_Tab4) und ihrer Soziodemografie (Zusatzmaterial online: Sup3_Tab5) unterscheiden lassen.

Personen aus **Cluster 1** wiesen die niedrigsten Werte im AFCCQ-Gesamtscore (−6,35) sowie in nahezu allen Bereichen auf. Diese Gruppe umfasste überwiegend Frauen, mit dem höchsten Anteil an Personen über 85 Jahren (26,7 %). Viele lebten allein (42,6 %) oder in Sozialwohnungen (6,9 %), bei gleichzeitig niedrigem Bildungsniveau. Cluster 1 wies die größte Gruppe von Personen mit mindestens einer chronischen Erkrankung (69,3 %) auf und verzeichnete die niedrigste Bewertung der Lebensqualität (5,8).

Im Gegensatz dazu wiesen Personen aus **Cluster 2** moderat bessere Werte in urbaner Zufriedenheit (AFCCQ-Gesamtscore 23,10) auf. Diese Gruppe war etwas jünger und umfasste mehr Personen mit höherem Bildungsniveau. Ein Großteil lebte in Eigenheimen (66,9 %), was sich in einem höheren Wert für Wohnen (5,99) widerspiegelte. Personen in Cluster 2 hatten ebenfalls häufig mindestens eine chronische Erkrankung (61,6 %) und auch ihre Lebensqualität (7,1) war niedriger bewertet.

Personen aus **Cluster 3** erreichten einen AFCCQ-Gesamtscore von 43,32. Diese Gruppe zeigte höhere Werte in fast allen Bereichen. Diese Personen waren zufrieden, in guter gesundheitlicher Verfassung und profitierten von einem relativ hohen Bildungsniveau (44,4 %) sowie der häufigen Wohnsituation in Eigenheimen (70,1 %). Auch die Lebensqualität (7,9) war hoch. Diese Gruppe hatte einen stabilen Zugang zu Ressourcen und nahm am städtischen Leben aktiv teil.

Personen aus **Cluster 4** erzielten den höchsten AFCCQ-Gesamtscore (66,06) und wiesen hohe Werte in nahezu allen Bereichen auf. Diese Gruppe hatte den geringsten Anteil an hochaltrigen Personen (≥ 85 Jahre) und den höchsten Anteil an Personen mit hoher Bildung (48,2 %) und Eigenheimbesitz (75,0 %). Auch die Einschätzung von Lebensqualität war mit 8,7 am höchsten.

## Diskussion

Die vorliegende Studie stellte den Prozess und die Ergebnisse einer Übersetzung, transkulturellen Adaptation, psychometrischen Evaluation und Cluster-Analyse der deutschen Version des AFCCQ vor. Die Ergebnisse zeigen, dass der AFCCQ-DE ein valides und zuverlässiges Instrument zur Bewertung der Altersfreundlichkeit von Städten und Gemeinden ist. Die Validierung dieses Tools ermöglicht nun sowohl Baseline-Erhebungen als auch national und internationale Vergleiche der Altersfreundlichkeit von Städten und Gemeinden im deutschsprachigen Raum. Die mit dem AFCCQ erhobenen Daten spiegeln die Meinungen und Erfahrungen älterer Menschen wider: Die Umfrage in Oldenburg brachte Erkenntnisse darüber, wie ältere Menschen die WHO-Bereiche bezüglich Altersfreundlichkeit erleben. In der Befragung in Oldenburg wurde der Bereich *Wohnen* von den Befragten im Vergleich zu den anderen 8 Bereichen am positivsten bewertet, was auf sichere Wohnverhältnisse hinweist. Dies bestätigt die Bedeutung von barrierefreien und zugänglichen Wohnlösungen, die den Bedürfnissen älterer Menschen Rechnung tragen. Anpassungen bestehender Wohnräume, die Schaffung von altersgerechten und barrierefreien Neubauten sowie die Entwicklung integrativer Quartiere sind wesentliche Maßnahmen, um die Selbstständigkeit älterer Menschen zu fördern [27]. Solche Maßnahmen tragen auch generationenübergreifend zu einer Verbesserung der Wohnqualität bei [9, 10]. *Respekt und soziale Einbindung* als wesentliche, diskriminierungssensible Aspekte altersfreundlicher Umfelder wurden ebenfalls positiv erlebt. Auch die *öffentlichen Verkehrsmittel* erhielten positive Bewertungen, was auf eine gute Erreichbarkeit und möglicherweise bereits umgesetzte erfolgreiche kommunale Strategien hinweist. Effiziente und zugängliche Mobilitätslösungen sind essenziell für die soziale Teilhabe älterer Menschen und ein zentraler Bestandteil altersfreundlicher Städte [5].

Dagegen wurde der Bereich *kommunale Unterstützung und Gesundheitsversorgung* schlechter bewertet, was auf Defizite in der Verfügbarkeit und Zugänglichkeit entsprechender Angebote hinweist. Eine umfassende und anpassungsfähige Gesundheitsversorgung ist entscheidend, um den vielfältigen Bedürfnissen einer älteren, aber heterogenen Bevölkerung gerecht zu werden [4, 5]. Verbesserungen in diesem Bereich sind zentral, um älteren Menschen ein selbstbestimmtes Leben zu ermöglichen [28].

Der Bereich *öffentlicher Raum und die Gebäude* schnitten ebenfalls weniger positiv ab, insbesondere in Bezug auf Barrierefreiheit und Infrastrukturqualität. Themen wie die Instandhaltung von Gehwegen und die Schaffung barrierefreier öffentlicher Räume fördern nicht nur die Mobilität, sondern auch die soziale Teilhabe und Lebensqualität älterer Menschen [5, 8, 11, 12].

Die Daten zeigen, ähnlich wie in anderen europäischen Städten [20, 29–31], 4 Cluster älterer Personen mit unterschiedlichen Wahrnehmungen, Erwartungen und Bedürfnissen im städtischen Raum. Cluster 1 repräsentiert die vulnerabelste Gruppe mit den größten Herausforderungen, während Cluster 4 von individuellen Ressourcen profitiert und die Stadt als besonders altersfreundlich empfindet. Cluster 4 schien aufgrund der Lebenslage, objektive Defizite besser zu kompensieren und auch weniger auf altersfreundliche Strukturen angewiesen zu sein. Eine inklusive altersfreundliche Stadtentwicklung sollte deshalb ihre Anstrengungen und Ressourcen gezielt auf weniger privilegierte Gruppen richten. Die Cluster zeigen somit, dass „Aging in Place“ in Städten und Gemeinden von Ungleichheit, Gemeinschaft und Ressourcen beeinflusst wird [3], und es wichtig ist, dass Interventionen an unterschiedliche Lebensphasen im Alter und Zielgruppen angepasst werden [31].

Gleichzeitig sind bei der Interpretation der Ergebnisse folgende Punkte zu beachten: Obwohl die Stichprobe in Oldenburg zufällig gezogen wurde, erfolgte keine Schichtung nach zusätzlichen Kriterien wie Alter, Geschlecht oder sozioökonomischem Status. Dies könnte dazu führen, dass bestimmte Gruppen in der Stadtbevölkerung unter- oder überrepräsentiert sind. Die Universität verfügt nicht über Adressinformationen der angeschriebenen Personen. Dies bedeutet, dass sie nicht feststellen kann, ob bestimmte Stadtteile möglicherweise weniger häufig angeschrieben wurden und somit in den Ergebnissen unterrepräsentiert sind. Personen mit Sprachbarrieren, schlechter Sehfähigkeit oder anderen Einschränkungen hatten möglicherweise Schwierigkeiten, an der Studie teilzunehmen. Weiterhin waren Bewohner*innen von Pflegeheimen nicht in die Erhebung eingeschlossen. Qualitative Erhebungen könnten helfen, die AFCCQ-DE-Ergebnisse besser zu interpretieren, indem sie subjektive Erfahrungen und das Zusammenspiel räumlicher sowie sozialer Faktoren beleuchten.

## Fazit für die Praxis

Die Altersfreundlichkeit von Städten sollte systematisch aus Sicht älterer Menschen erfasst werden. Der AFCCQ-DE bietet eine verlässliche Grundlage für quantitative und vergleichbare Indikatoren, um die individuellen Erfahrungen und Bedürfnisse besser zu verstehen.

Die Entwicklung altersfreundlicher Städte erfordert enge Kooperationen zwischen verschiedenen Akteuren. Wissenschaftliche Analysen, praktische Erfahrung und die Einbeziehung älterer Menschen können helfen, bedarfsgerechte Lösungen zu entwickeln.

Altersfreundlichkeit ist ein dynamischer Prozess, der kontinuierlich überprüft und weiterentwickelt werden sollte. Ergänzende qualitative Methoden bieten tiefere Einblicke und ermöglichen eine differenzierte Bewertung.

## Supplementary Information

Supplement 1 – AFCCQ-German language

Supplement 2 – Fragebogenanpassungen

Supplement 3 – Ergebnistabellen

Supplement 4 – Einsatz des AFCCQ in anderen Ländern

## Data Availability

Auf begründete Anfrage können die Daten in anonymisierter Form eingesehen werden.
