# Supplementary material for: How age-friendly are cities and communities? German version of the Age-Friendly Cities and Communities Questionnaire (AFCCQ)
Source: Z Gerontol Geriatr. 2025 Apr 30;58(8):668–73. [Article in German] doi: 10.1007/s00391-025-02440-6 (PMC12644111; doi:10.1007/s00391-025-02440-6)
Supplement: Supplementary file 1 — Supplement 1 – AFCCQ-German language [file 391_2025_2440_MOESM1_ESM.pdf]

The Age-Friendly Cities and Communities Questionnaire AFCCQ (German)

Item	Bereich	Stimme überhaupt nicht zu	Stimme nicht zu	Weder noch	Stimme zu	Stimme voll und ganz zu
	**WOHNEN**					
Q1	Mein Zuhause ist für mich gut zugänglich					
Q2	Mein Zuhause ist für Personen, die mich besuchen, gut zugänglich					
	**SOZIALE TEILHABE**					
Q3	In meinem Wohnviertel gibt es genügend Gelegenheiten, anderen Menschen zu begegnen					
Q4	Aktivitäten und Veranstaltungen finden an Orten statt, die für mich erreichbar sind					
Q5	Informationen über Aktivitäten und Veranstaltungen sind ausreichend vorhanden und für mich geeignet.					
Q6	Ich finde das Angebot an Veranstaltungen und Aktivitäten abwechslungsreich genug					
	**RESPEKT UND SOZIALE EINBINDUNG**					
Q7*	Ich bekomme manchmal unangenehme oder unangemessene Kommentare wegen meines Alters					
Q8*	Ich werde manchmal wegen meines Alters benachteiligt					
	**BÜRGERSCHAFTLICHES ENGAGEMENT UND BESCHÄFTIGUNG**					
Q9	Ich habe genügend Gelegenheiten, mich mit jüngeren Generationen auszutauschen					
Q10	Ich fühle mich als ein wertgeschätztes Mitglied der Gesellschaft					
	**KOMMUNIKATION UND INFORMATION**					
Q11	Informationen der Stadt/Gemeinde und anderer sozialer Angebote, gedruckt wie online, sind in Bezug auf Schriftart und Buchstabengröße leicht zu lesen					
Q12	Informationen der Stadt/Gemeinde und anderer sozialer Angebote, gedruckt wie online, sind in verständlicher Sprache verfasst					
	**KOMMUNALE UNTERSTÜTZUNG UND GESUNDHEITSVERSORGUNG**					
Q13	Das Angebot an Sozial- und Gesundheitsdienstleistungen in meiner Stadt/Gemeinde ist für mich ausreichend					
Q14	Wenn ich krank bin, bekomme ich die Gesundheitsversorgung und Hilfe, die ich brauche					
Q15	Wenn es notwendig ist, kann ich Sozial- und Gesundheitsdienstleistungen telefonisch und persönlich leicht erreichen					
Q16	Ich habe genügend Informationen über Sozial- und Gesundheitsdienstleistungen in meinem Wohnviertel					
Q17	Das Personal im Bereich der Sozial- und Gesundheitsdienstleistungen ist respektvoll genug					
	**ÖFFENTLICHER RAUM UND GEBÄUDE**					
Q18	In meinem Wohnviertel kann man sich mit Rollator oder Rollstuhl gut genug bewegen					
Q19	Die Geschäfte in meinem Wohnviertel sind mit einem Rollator oder Rollstuhl gut genug zugänglich					
	**ÖFFENTLICHE VERKEHRSMITTEL**					
Q20	Ich kann in meinem Wohnviertel ohne Schwierigkeiten in den öffentlichen Nahverkehr einsteigen					
Q21	Der öffentliche Nahverkehr in meinem Wohnviertel ist ohne Schwierigkeiten zu erreichen und zu nutzen					
	**FINANZIELLE SITUATION**					
Q22	Mein Einkommen reicht ohne Probleme aus, meine Grundbedürfnisse abzudecken					
Q23	Ich kann von meinem Einkommen gut leben					

The Age-Friendly Cities and Communities Questionnaire AFCCQ (German)

INTERPRETATION AFCCQ GESAMTPUNKTZAHL UND EINZELNE BEREICHE

	----	---	--	-	+	++	+++	++++
**AFCCQ Total score**	**≤ -35.1**	**-23.1 – -35.0**	**-11.5 – -23.0**	**-11.4 – 0.0**	**0.1 – 11.4**	**11.5 – 23.0**	**23.1 – 35.0**	**≥ 35.1**
Wohnen	≤ -3.1	-2.1 – -3.0	-1.1 – -2.0	-1.0 – 0.0	0.1 – 1.0	1.1 – 2.0	2.1 – 3.0	≥ 3.1
Soziale Teilhabe	≤ -6.1	-4.1 – -6.0	-2.1 – -4.0	-2.0 – 0.0	0.1 – 2.0	2.1 – 4.0	4.1 – 6.0	≥ 6.1
Respekt und soziale Einbindung	≤ -3.1	-2.1 – -3.0	-1.1 – -2.0	-1.0 – 0.0	0.1 – 1.0	1.1 – 2.0	2.1 – 3.0	≥ 3.1
Bürgerschaftliches Engagement und Beschäftigung	≤ -3.1	-2.1 – -3.0	-1.1 – -2.0	-1.0 – 0.0	0.1 – 1.0	1.1 – 2.0	2.1 – 3.0	≥ 3.1
Kommunikation und Information	≤ -3.1	-2.1 – -3.0	-1.1 – -2.0	-1.0 – 0.0	0.1 – 1.0	1.1 – 2.0	2.1 – 3.0	≥ 3.1
Kommunale Unterstützung und Gesundheitsversorgung	≤ -7.6	-5.1 – -7.5	-2.6 – -5.0	-2.5 – 0.0	0.1 – 2.5	2.6 – 5.0	5.1 – 7.5	≥ 7.6
Öffentlicher Raum und Gebäude	≤ -3.1	-2.1 – -3.0	-1.1 – -2.0	-1.0 – 0.0	0.1 – 1.0	1.1 – 2.0	2.1 – 3.0	≥ 3.1
Öffentliche Verkehrsmittel	≤ -3.1	-2.1 – -3.0	-1.1 – -2.0	-1.0 – 0.0	0.1 – 1.0	1.1 – 2.0	2.1 – 3.0	≥ 3.1
Finanzielle Situation	≤ -3.1	-2.1 – -3.0	-1.1 – -2.0	-1.0 – 0.0	0.1 – 1.0	1.1 – 2.0	2.1 – 3.0	≥ 3.1

Punktesystem

Alle Fragen des AFCCQ werden auf einer 5-Punkte-Skala beantwortet, die von "stimme überhaupt nicht zu" bis " stimme voll und ganz zu" reicht.

Punktzahlen: -2 = stimme überhaupt nicht zu; -1 = stimme nicht zu; 0 = weder noch; 1 = stimme zu; 2 = stimme völlig zu.

Die mit einem Sternchen (*) gekennzeichneten Items sollten in umgekehrter Richtung umcodiert werden (-2=2, -1=1, 0=0, 1=-1, 2=-2)

Addieren Sie alle Punkte des AFCCQ für die Gesamtpunktzahl.

Addieren Sie alle Punktzahlen der einzelnen Bereiche, um die bereichsspezifische Punktzahl zu erhalten.

Impressum

Dieser Fragebogen wurde erstellt von: Prof. Dr. Kathrin Boerner (Carl von Ossietzky Universität Oldenburg), Adele Grenz, M.Sc. (Carl von Ossietzky Universität Oldenburg), Dr. Michael Weinhardt (Deutsches Zentrum für Alternsfragen), Prof. Dr. Moritz Hess (Hochschule Niederrhein, Prof. Dr. Joost van Hoof (The Hague University of Applied Sciences), Dr. Jeroen Dikken (The Hague University of Applied Sciences), mit Unterstützung der Stadt Oldenburg und der Körber-Stiftung.

CC BY

ISBN: 9789083442075

Carl von Ossietzky Universität Oldenburg

In Kooperation mit Stadt Oldenburg

Körber Stiftung

THE HAGUE UNIVERSITY OF APPLIED SCIENCES
