# Supplementary material for: How age-friendly are cities and communities? German version of the Age-Friendly Cities and Communities Questionnaire (AFCCQ)
Source: Z Gerontol Geriatr. 2025 Apr 30;58(8):668–73. [Article in German] doi: 10.1007/s00391-025-02440-6 (PMC12644111; doi:10.1007/s00391-025-02440-6)
Supplement: Supplementary file 2 — Supplement 2 – Fragebogenanpassungen [file 391_2025_2440_MOESM2_ESM.pdf]

Supplement 2 – Wie altersfreundlich sind Städte und Gemeinden? Deutsche Version des Age-Friendly Cities and Communities Questionnaire (AFCCQ)

Tabelle 1: Übersicht der Fragebogenanpassungen

Bereich	Fragen Englisch	Übersetzung 1 (Deutsch)	Anpassungen finale Version
WOHNEN	Q1 My house is accessible to me	Mein Zuhause ist für mich gut zugänglich.	*Die Formulierung wurde nicht verändert.*
	Q2 My house is accessible to the people who come to visit me	Mein Zuhause ist für Personen, die mich besuchen, gut zugänglich.	*Die Formulierung wurde nicht verändert.*
SOZIALE TEILHABE	Q3 There are enough opportunities to meet people in my neighbourhood	In meinem Wohnviertel gibt es genügend Gelegenheiten, anderen Menschen zu begegnen.	*Die Formulierung wurde nicht verändert.*
	Q4 Activities and events are organised in places that are accessible to me	Aktivitäten und Veranstaltungen finden an Orten statt, die für mich gut zugänglich sind.	*Die Formulierung wurde geändert zu:* **„die für mich erreichbar sind."**
	Q5 The information about activities and events is enough for me and also suitable for me	Informationen über Aktivitäten und Veranstaltungen sind ausreichend vorhanden und passen auch für mich.	*Die Formulierung wurde angepasst zu:* **„und für mich geeignet."**
	Q6 I find the range of events and activities sufficiently varied	Ich finde das Angebot an Veranstaltungen und Aktivitäten ist ausreichend abwechslungsreich.	**„Ausreichend abwechslungsreich"** *wurde zu* **„abwechslungsreich genug"** *geändert.*
RESPEKT UND SOZIALE EINBINDUNG	Q7 I sometimes get annoying or negative remarks because of my age	Ich bekomme manchmal unangenehme oder unangemessene Kommentare wegen meines Alters.	*Die Formulierung wurde nicht verändert.*
	Q8 I sometimes face discrimination because of my age	Ich werde manchmal wegen meines Alters diskriminiert.	**„Diskriminiert"** *wurde durch* **„benachteiligt"** *ersetzt.*
BÜRGERSCHAFTLICHES ENGAGEMENT UND BESCHÄFTIGUNG	Q9 I have enough opportunities to interact with younger generations	Ich habe genügend Gelegenheiten, mich mit jüngeren Generationen auszutauschen.	*Die Formulierung wurde nicht verändert.*
	Q10 I feel like a valued member of society	Ich fühle mich wie ein wertgeschätztes Mitglied der Gesellschaft.	**„Wie ein wertgeschätztes Mitglied"** *wurde zu* **„als ein wertgeschätztes Mitglied"** *geändert.*

Bereich	Fragen Englisch	Übersetzung 1 (Deutsch)	Anpassungen finale Version
KOMMUNIKATION UND INFORMATION	Q11 Printed and digital information from the municipality and other social institutions is easy to read in terms of font and size	Informationen der Gemeinde und anderer sozialer Angebote, gedruckt wie online, sind in Bezug auf Schriftart und Buchstabengröße leicht zu lesen.	**„Informationen der Gemeinde“** *wurde zu* **„Informationen der Stadt/Gemeinde“** *geändert.*
	Q12 Printed and digital information from the municipality and other social institutions is written in understandable language	Informationen der Gemeinde und anderer sozialer Angebote, gedruckt wie online, sind in verständlicher Sprache verfasst.	**„Informationen der Gemeinde“** *wurde zu* **„Informationen der Stadt/Gemeinde“** *geändert.*
KOMMUNALE UNTERSTÜTZUNG UND GESUNDHEITSVERSORGUNG	Q13 The supply of care and welfare in my city is enough for me	Das Angebot an Pflege und sozialen Dienstleistungen in meiner Stadt ist für mich ausreichend.	**„Pflege und sozialen Dienstleistungen“** *wurde zu* **„Sozial- und Gesundheitsdienstleistungen“** *geändert.*
	Q14 When I am ill, I receive the care and help I need	Wenn ich krank bin, bekomme ich die Pflege und Hilfe, die ich brauche.	**„Pflege und Hilfe“** *wurde durch* **„Gesundheitsversorgung und Hilfe“** *ersetzt.*
	Q15 If necessary, I can easily reach care and welfare services by telephone and in person	Wenn es notwendig ist, kann ich Pflege- und soziale Dienstleistungen telefonisch und persönlich leicht erreichen.	**„Pflege- und soziale Dienstleistungen“** *wurde durch* **„Sozial- und Gesundheitsdienstleistungen“** *ersetzt.*
	Q16 I have enough information about care and welfare services in my neighbourhood	Ich habe genügend Informationen über Pflege- und soziale Dienstleistungen in meinem Wohnviertel.	**„Pflege- und soziale Dienstleistungen“** *wurde durch* **„Sozial- und Gesundheitsdienstleistungen“** *ersetzt.*
	Q17 Care and welfare workers in my neighbourhood are sufficiently respectful	Das Personal im Bereich der Pflege und sozialen Dienstleistungen ist ausreichend respektvoll.	**„Pflege und sozialen Dienstleistungen“** *wurde durch* **„Sozial- und Gesundheitsdienstleistungen“** *ersetzt.*
ÖFFENTLICHER RAUM UND GEBÄUDE	Q18 My neighbourhood is sufficiently accessible for a wheeled walker or wheelchair	Ich kann mich in meinem Wohnviertel mit Rollator oder Rollstuhl ausreichend gut bewegen.	*Die Formulierung wurde geändert zu:* **„In meinem Wohnviertel kann man sich mit Rollator oder Rollstuhl gut genug bewegen.“**

Bereich	Fragen Englisch	Übersetzung 1 (Deutsch)	Anpassungen finale Version
	Q19 The shops in my neighbourhood are sufficiently accessible with a wheeled walker or wheelchair	Die Geschäfte in meinem Wohnviertel sind mit einem Rollator oder Rollstuhl ausreichend gut zugänglich.	*Die Formulierung wurde geändert zu:* **„Die Geschäfte in meinem Wohnviertel sind mit einem Rollator oder Rollstuhl gut genug zugänglich."**
ÖFFENTLICHE VERKEHRSMITTEL	Q20 I can easily get on the bus or tram in my neighbourhood	Ich kann in meinem Wohnviertel ohne Schwierigkeiten in den Bus oder die Straßenbahn einsteigen.	**„Den Bus oder die Straßenbahn"** *wurde durch* **„den öffentlichen Nahverkehr"** *ersetzt.*
	Q21 The bus and tram stops in my neighbourhood are easy to reach and use	Die Bus- und Straßenbahnhaltestellen in meinem Wohnviertel sind ohne Schwierigkeiten zu erreichen und zu nutzen.	**„Bus- und Straßenbahnhaltestellen"** *wurde durch* **„den öffentlichen Nahverkehr"** *ersetzt.*
FINANZIELLE SITUATION	Q22 My income is sufficient to cover my basic needs without any problems	Mein Einkommen reicht ohne Probleme aus, meine Grundbedürfnisse abzudecken.	*Die Formulierung wurde nicht verändert.*
	Q23 I live well on my income	Ich kann von meinem Einkommen gut leben.	*Die Formulierung wurde nicht verändert.*
