# Supplementary material for: How age-friendly are cities and communities? German version of the Age-Friendly Cities and Communities Questionnaire (AFCCQ)
Source: Z Gerontol Geriatr. 2025 Apr 30;58(8):668–73. [Article in German] doi: 10.1007/s00391-025-02440-6 (PMC12644111; doi:10.1007/s00391-025-02440-6)
Supplement: Supplementary file 3 — Supplement 3 – Ergebnistabellen [file 391_2025_2440_MOESM3_ESM.pdf]

Supplement 3 – Wie altersfreundlich sind Städte und Gemeinden? Deutsche Version des Age-Friendly Cities and Communities Questionnaire (AFCCQ)

Tab. 1 Phase 2: Adaptation I-CVI face- und content-validity AFCCQ-Items

**Item**	**Itemtext**	**Oldenburg**	
		Face I-CVI	Content I-CVI
	**Wohnen**		
Q1	Mein Haus ist für mich gut zugänglich.	1,00	1,00
Q2	Mein Haus ist für Personen, die mich besuchen, gut zugänglich.	0,88	0,80
	**Soziale Teilhabe**		
Q3	In meinem Wohnviertel gibt es genügend Gelegenheiten, anderen Menschen zu begegnen.	1,00	0,90
Q4	Aktivitäten und Veranstaltungen finden an Orten statt, die für mich erreichbar sind.	0,94	1,00
Q5	Informationen über Aktivitäten und Veranstaltungen sind ausreichend vorhanden und für mich geeignet.	1,00	0,89
Q6	Ich finde da Angebot an Veranstaltungen und Aktivitäten abwechslungsreich genug.	0,94	0,89
	**Respekt und soziale Einbindung**		
Q7	Ich bekomme manchmal unangenehme oder unangemessene Kommentare wegen meines Alters.	0,63	0,40
Q8	Ich werde manchmal wegen meines Alters diskriminiert.	0,75	0,50
	**Bürgerschaftliches Engagement und Beschäftigung**		
Q9	Ich habe genügend Gelegenheiten, mich mit jüngeren Generationen auszutauschen.	0,88	0,70
Q10	Ich fühle mich als wertgeschätztes Mitglied der Gesellschaft.	0,81	0,50
	**Kommunikation und Information**		
Q11	Informationen der Stadt/Gemeinde und anderer sozialer Angebote, gedruckt wie online, sind in Bezug auf Schriftart und Buchstabengröße leicht zu lesen.	0,94	0,70
Q12	Informationen der Stadt/Gemeinde und anderer sozialer Angebote, gedruckt wie online, sind in verständlicher Sprache verfasst.	0,94	0,70
	**Kommunale Unterstützung und Gesundheitsversorgung**		
Q13	Das Angebot an Sozial- und Gesundheitsdienstleistungen in meiner Stadt/Gemeinde ist für mich ausreichend.	1,00	0,90
Q14	Wenn ich krank bin, bekomme ich die Gesundheitsversorgung und Hilfe, die ich brauche.	1,00	0,80
Q15	Wenn es notwendig ist, kann ich Sozial- und Gesundheitsdienstleistungen telefonisch und persönlich leicht erreichen.	1,00	0,90
Q16	Ich habe genügend Informationen über Sozial- und Gesundheitsdienstleistungen in meinem Wohnviertel.	1,00	1,00
Q17	Das Personal im Bereich der Sozial- und Gesundheitsdienstleistungen ist respektvoll genug.	1,00	0,67
	**Öffentlicher Raum und Gebäude**		
Q18	In meinem Wohnviertel kann man sich mit Rollator oder Rollstuhl gut genug bewegen.	0,94	0,90
Q19	Die Geschäfte in meinem Wohnviertel sind mit einem Rollator oder Rollstuhl gut genug zugänglich.	0,88	1,00
	**Öffentliche Verkehrsmittel**		
Q20	Ich kann in meinem Wohnviertel ohne Schwierigkeiten in den öffentlichen Nahverkehr einsteigen.	1,00	1,00
Q21	Der öffentliche Nahverkehr in meinem Wohnviertel ist ohne Schwierigkeiten zu erreichen und zu nutzen.	1,00	1,00
	**Finanzielle Situation**		
Q22	Mein Einkommen reicht ohne Probleme aus, meine Grundbedürfnisse abzudecken.	1,00	0,67
Q23	Ich kann von meinem Einkommen gut leben.	1,00	0,67

Tab. 2 Phase 3 Validierung: Anpassung der Daten aus Deutschland an das Originalmodell von Dikken et al. 2020

Modell	Normed X2	Degrees of freedom	Comparative Fit Index (CFI)	Tucker Lewis Index (TLI)	Root-Mean Squared Residual (SRMR)	Root-Mean Square Error of Approximation (RMSEA) [90% CI]
Modell 1.	3.340	194	0.948	0.932	0.0423	0.058 [0.053 – 0.063]

Ein Modell, das neun Bereiche abdeckt, wurde erstellt. Die Modellanpassungsstatistiken für Modell 1 deuten auf eine insgesamt gute Passung der Daten hin. Der normierte X^2-Wert von 3,340 liegt im akzeptablen Bereich, was darauf hindeutet, dass das Modell die beobachteten Daten gut beschreibt, während es eine Balance zwischen Komplexität und Sparsamkeit wahrt. Der Comparative Fit Index (CFI) von 0,948 und der Tucker Lewis Index (TLI) von 0,932 übertreffen beide den üblichen Schwellenwert von 0,90 und zeigen eine starke Passung im Vergleich zu einem Basismodell, was die Angemessenheit der Modellstruktur unterstützt. Der Root-Mean Squared Residual (SRMR)-Wert von 0,0423 liegt deutlich unter dem empfohlenen Cutoff von 0,08 und zeigt, dass die vorhergesagten und beobachteten Korrelationen des Modells nahe beieinander liegen, was die gute Modellanpassung weiter unterstützt. Der Root-Mean Square Error of Approximation (RMSEA) beträgt 0,058, mit einem 90%-Konfidenzintervall von [0,053 – 0,063]. Dieser Wert liegt im akzeptablen Bereich (unter 0,06 bis 0,08) und deutet auf eine relativ gute Annäherung des Modells an die Populations-Daten hin. Insgesamt deuten die Fit-Indizes darauf hin, dass Modell 1 eine starke und akzeptable Passung zu den Daten bietet.

Tab. 3 Phase 3 Validierung: Reliabilität pro Bereich des AFCCQ-DE

**Bereich**	**Wohnen**	**Soziale Teilhabe**	**Respekt und soziale Einbindung**	**Bürgerschaftliches Engagement und Beschäftigung**	**Kommunikation und Information**	**Kommunale Unterstützung und Gesundheitsversorgung**	**Öffentlicher Raum und Gebäude**	**Öffentliche Verkehrsmittel**	**Finanzielle Situation**
**Zusammengefasste Reliabilität**	0.839	0.865	0.808	0.683	0.776	0.849	0.798	0.900	0.956

Die Kompositreliabilität für die 9 Subskalen liegt zwischen 0,683 und 0,956 (siehe Tabelle 3). Insbesondere die beiden Skalen („Öffentliche Verkehrsmittel“ und „Finanzielle Situation“) zeigen mit CR-Werten von 0,900 bzw. 0,956 eine hohe interne Konsistenz.

Teilnehmende und fehlende Daten

Insgesamt nahmen 905 Personen im Alter von 65 Jahren und älter an der Studie teil. Davon wurden 23 Teilnehmende von der weiteren Analyse ausgeschlossen, da sie keine vollständigen Angaben zu allen zentralen demografischen Variablen machten, darunter Alter, Geschlecht, Bildungsgrad gesundheitsbezogene Variablen und die selbst eingeschätzte Lebensqualität. Diese demografischen Variablen waren für den Imputationsprozess und die anschließende Clusteranalyse unerlässlich, da sie als wichtige Prädiktoren dienen. Da eine zuverlässige Imputation der fehlenden Daten für diese Teilnehmenden nicht möglich war, wurden sie aus der Analyse ausgeschlossen. Der Ausschluss von 23 Personen, was etwa 2,5 % der Gesamtstichprobe entspricht, dürfte keine signifikante Verzerrung verursachen. Die verbleibende Stichprobe von 882 Personen wurde als ausreichend groß für die Analyse angesehen.

Von den verbleibenden 882 Teilnehmenden hatten 674 vollständige Daten zu allen demografischen Variablen, während bei 208 Personen Werte in mindestens einer dieser Variablen fehlten, was 2,4 % der Daten entspricht. In Bezug auf die AFCCQ-Skala hatten 687 Personen alle Items vollständig beantwortet, während bei 195 Personen mindestens ein Wert fehlte, was 2,6 % der Daten ausmacht. Die fehlenden Daten für sowohl demografische Merkmale als auch AFCCQ-Items wurden als zufällig fehlend („Missing at Random“, MAR) klassifiziert, was eine Imputation als angemessen erscheinen ließ.

Imputationsverfahren

Zur Behandlung der fehlenden Daten wurde zunächst eine Mehrfachimputation in Betracht gezogen. Aufgrund des geringen Anteils an fehlenden Daten und der Komplexität des Datensatzes wurde jedoch stattdessen eine Einzelimputation durchgeführt. Die Imputation erfolgte in SPSS (Version 27) mithilfe der Markov-Chain-Monte-Carlo-Methode (MCMC). Obwohl die AFCCQ-Items ordinal skaliert sind, wurden sie während der Imputation als metrische Variablen behandelt, um eine Konvergenz des Modells zu gewährleisten. Diese Umwandlung wurde als angemessen betrachtet, da die Abstände zwischen den ordinalen Kategorien hinreichend konsistent waren. Fehlende Werte wurden mit der Methode des Predictive Mean Matching (PMM) imputiert, wobei realistische Werte aus den fünf nächstliegenden vorhergesagten Fällen ausgewählt wurden. Es wurde ein einzelner imputierter Datensatz mit maximal 20 Iterationen generiert, um die Unsicherheit im Imputationsprozess zu berücksichtigen. Die Toleranz für Singularität wurde auf 1E-008 gesetzt, um Multikollinearität unter den Prädiktorvariablen zu kontrollieren.

Clusteranalyse des AFCCQ

Zur Identifikation unterschiedlicher Gruppen innerhalb der Stichprobe wurde eine Clusteranalyse durchgeführt. Dabei wurden zwei Stufen der Clusteranalyse angewendet, basierend auf den Empfehlungen von Milligan (1980). Die normalisierten AFCCQ-Bereiche dienten als Grundlage zur Bestimmung der Ähnlichkeiten zwischen den Teilnehmenden. Zunächst wurde eine agglomerative hierarchische Clusteranalyse (HCA) mit der Ward-Methode und quadrierter euklidischer Distanz verwendet, um die optimale Anzahl an Clustern zu bestimmen. Anschließend wurde die Stichprobe in zwei Teile geteilt, um die Clusteranzahl zu validieren. Zudem wurde der Bonferroni-Test durchgeführt, um die statistische Signifikanz der Unterschiede zwischen den Clustern zu prüfen und Fehler des Typs I aufgrund multipler Tests zu kontrollieren (Holm, 1979).

Nach der Identifikation stabiler Cluster durch HCA wurde eine k-Means-Clusteranalyse durchgeführt, um die Gruppen basierend auf den AFCCQ-Bereiche zu klassifizieren und zu interpretieren. Danach wurden die demografischen Merkmale innerhalb der Cluster analysiert, um Typologien zu entwickeln. Alle Analysen wurden mit SPSS Version 29.0 (IBM Corp., 2021) durchgeführt.

Clusterbestimmung

Zur Identifizierung der optimalen Clusteranzahl wurde ein Dendrogramm erstellt. Der Clustering-Prozess deutete auf drei, vier oder sechs sinnvolle Cluster hin. Zur Validierung der Lösung wurde die Stichprobe halbiert und die hierarchische Clusteranalyse (HCA) erneut durchgeführt, wobei sich die gleichen Ergebnisse zeigten. Der Bonferroni-Test ergab, dass für die Vier-Cluster-Lösung alle p-Werte signifikant waren, was auf deutliche Unterschiede zwischen den Clustern hinweist. Bei der Sechs-Cluster-Lösung zeigten mehrere Bereiche keine signifikanten Unterschiede, und ein Cluster war mit nur 16 Teilnehmenden sehr klein. Daher wurde die Vier-Cluster-Lösung gewählt, da sie eine detaillierte und umfassende Analyse ermöglicht.

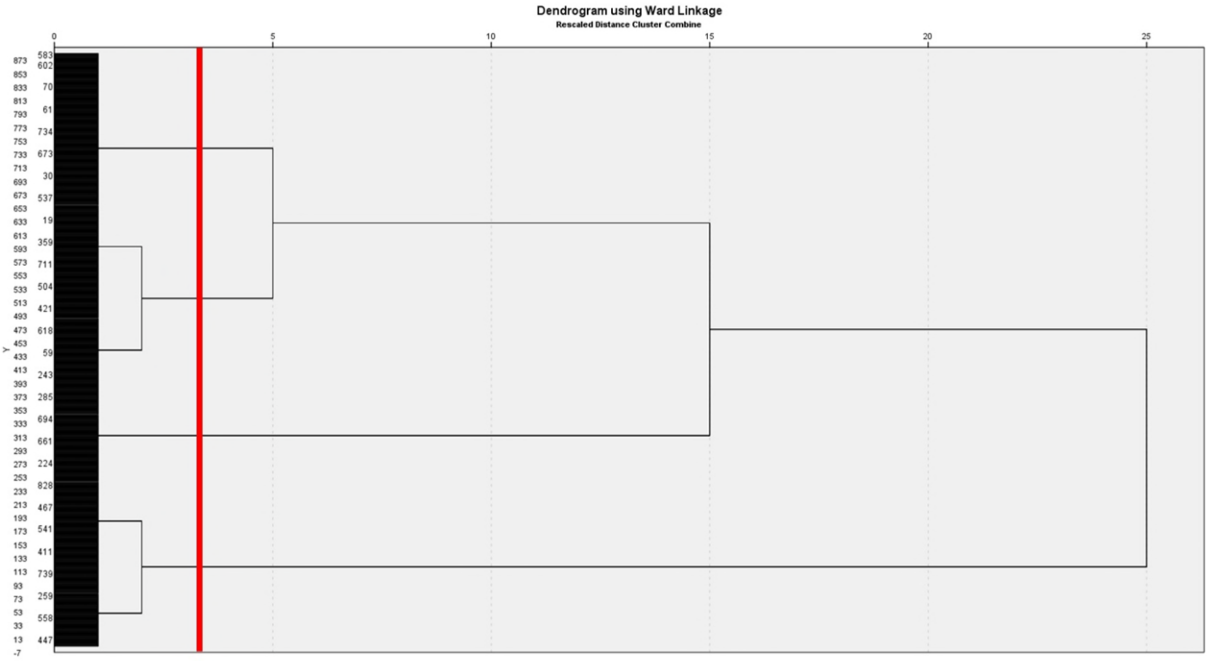

Abbildung 1. Dendrogramm, das die 3-, 4- oder 6-Cluster-Lösung zeigt. Die rote Linie markiert die Wahl der Vier-Cluster-Lösung.

Tab. 4 Cluster und normalisierte Clusterwerte des AFCCQ-DE (n=882)

**Bereich (-10 bis 10)**	**Cluster 1** n=101	**Cluster 2** n=229	**Cluster 3** n=324	**Cluster 4** n=228
Wohnen	3.42	5.99	7.62	9.47
	++	+++	++++	++++
Soziale Teilhabe	-2.25	1.51	4.72	7.04
	-	+	++	+++
Respekt und soziale Einbindung	-0.25	3.80	5.53	8.10
	-	++	+++	++++
Bürgerschaftliches Engagement und Beteiligung	-1.53	2.16	4.66	7.28
	-	+	++	+++
Kommunikation und Information	-0.64	1.95	4.51	6.92
	-	+	++	+++
Kommunale Unterstützung und Gesundheitsversorgung	-1.89	0.83	3.11	6.05
	-	+	++	+++
Öffentlicher Raum und Gebäude	-1.09	1.53	3.19	5.84
	-	+	++	+++
Öffentliche Verkehrsmittel	0.12	3.98	5.39	8.25
	+	++	+++	++++
Finanzielle Situation	-6.35	1.34	4.60	7.11
	---	+	++	+++
**AFCCQ-Gesamt (-90 bis +90)**	**-6.35**	**23.10**	**43.32**	**66.06**
	**-**	**++**	**++**	**+++**

Tab. 5 Soziodemographische Verteilung innerhalb der Cluster, n (%)				
	**Cluster 1** n=101	**Cluster 2** n=229	**Cluster 3** n=324	**Cluster 4** n=228
**Geschlecht, w**	64 (63.4)	133 (58.1)	168 (51.9)	117 (51.3)
**Alter**				
65-74 Jahre	47 (46.5)	115 (50.2)	163 (50.3)	114 (50.0)
75-84 Jahre	27 (26.7)	74 (32.3)	119 (36.7)	92 (40.4)
≥85 Jahre	27 (26.7)	40 (17.5)	42 (13.0)	22 (9.6)
**Geburtsland, DE**	93 (92.1)	206 (90.0)	312 (96.3)	211 (92.5)
**Bildungsgrad, ISCED**				
Niedrig	12 (11.9)	17 (7.4)	19 (5.9)	7 (3.1)
Mittel	60 (59.4)	116 (50.7)	161 (49.7)	111 (48.7)
Hoch	29 (28.7)	96 (41.9)	144 (44.4)	110 (48.2)
**Wohnsituation**				
Eigenheim	53 (52.5)	153 (66.9)	227 (70.1)	171 (75.0)
Miete	41 (40.6)	70 (30.6)	94 (29.0)	56 (24.6)
Sozialwohnung	7 (6.9)	6 (2.6)	3 (0.9)	1 (0.4)
**Allein lebend**	43 (42.6)	85 (37.1)	113 (34.9)	65 (28.5)
**Gesundheit**				
Erhält Unterstützung	46 (45.5)	77 (33.6)	87 (26.9)	71 (31.1)
Chronisch krank	70 (69.3)	141 (61.6)	136 (42.0)	90 (39.5)
Nutzung Rollator/Rollstuhl	41 (40.6)	51 (22.3)	46 (14.2)	20 (8.8)
**Lebensqualität, M (SD)** (Score 1 sehr niedrig-10 sehr hoch)	5.8 (1.8)	7.1 (1.4)	7.9 (1.3)	8.7 (1.1)
