# Supplementary material for: How age-friendly are cities and communities? German version of the Age-Friendly Cities and Communities Questionnaire (AFCCQ)
Source: Z Gerontol Geriatr. 2025 Apr 30;58(8):668–73. [Article in German] doi: 10.1007/s00391-025-02440-6 (PMC12644111; doi:10.1007/s00391-025-02440-6)
Supplement: Supplementary file 4 — Supplement 4 – Einsatz des AFCCQ in anderen Ländern [file 391_2025_2440_MOESM4_ESM.pdf]

Supplement 4 – Wie altersfreundlich sind Städte und Gemeinden? Deutsche Version des Age-Friendly Cities and Communities Questionnaire (AFCCQ)

Einsatz AFCCQ in anderen Ländern

- Netherlands [1]
- Turkey [2]
- Japan [3]
- Romania [4]
- North Macedonia [5]
- Israel [6]
- Poland [7]
- Australia [8]
- Russia [9]

References

1. Dikken J, van den Hoven RFM, van Staalduinen WH, Hulsebosch-Janssen LMT, van Hoof J. How Older People Experience the Age-Friendliness of Their City: Development of the Age-Friendly Cities and Communities Questionnaire. International Journal of Environmental Research and Public Health. 2020;17:6867. doi:10.3390/ijerph17186867.
2. Özer Z, Turan GB, Teke N. Age-friendly cities and communities questionnaire: A research on Turkish validity and reliability. Archives of Environmental & Occupational Health. 2023;78:38–47. doi:10.1080/19338244.2022.2061397.
3. Yamada K, Murotani K, Mano M, Lim Y, Yoshimatsu J. Age-Friendly Approach Is Necessary to Prevent Depopulation: Resident Architectural Designers and Constructors' Evaluation of the Age-Friendliness of Japanese Municipalities. International Journal of Environmental Research and Public Health 2023. doi:10.3390/ijerph20176626.
4. Ivan L, Dikken J, van Hoof J. Unveiling the experienced age-friendliness of older people in Bucharest: A comprehensive study using the validated Romanian age-friendly cities and communities questionnaire and cluster analysis. Habitat International. 2024;143:102973. doi:10.1016/j.habitatint.2023.102973.
5. Pavlovski D, Dikken J, Ollogu EB, van Hoof J. How older adults experience the age-friendliness of Skopje: Results of the validation of the AFCCQ for use in North Macedonia and a representative survey. Heliyon. 2024;10:e30372. doi:10.1016/j.heliyon.2024.e30372.
6. Ayalon L, Dikken J, van Hoof J. The Age-Friendly Cities and Communities Questionnaire: A validation study of the Hebrew version in Israel. Heliyon. 2024;10:e39182. doi:10.1016/j.heliyon.2024.e39182.
7. Perek-Białas JM, Skórska P, Maj M, Kazak JK, Dikken J, van Hoof J. The experienced age-friendliness in two Polish cities: An in-depth analysis of the views of older citizens. Habitat International. 2024;153:103201. doi:10.1016/j.habitatint.2024.103201.
8. Wasserman R, Barrie H, Dikken J, van Hoof J, Soebarto V. Validating the age-friendly cities and communities questionnaire in Australia: Revealing five distinct groups of older people in Greater Adelaide. Habitat International. 2025;156:103278. doi:10.1016/j.habitatint.2024.103278.
9. Ziganshina LE, Garaeva AF, Talipova LI, Khairullin RN, Dikken J, van Hoof J. Measuring the age-friendliness of cities in the Russian Federation: The translation, validation and application of the age-friendly cities and communities Questionnaire in the city of Kazan. Heliyon. 2025;11:e41100. doi:10.1016/j.heliyon.2024.e41100.
